# Repeatable negotiation rules? Only females show repeatable responses to partner removal in a brood-provisioning songbird

**DOI:** 10.1098/rsbl.2023.0136

**Published:** 2023-06-21

**Authors:** Davide Baldan, Matteo Beccardi, Manuel Fuertes-Recuero, Matteo Schiavinato, Lia Zampa, Andrea Pilastro, Alejandro Cantarero

**Affiliations:** ^1^ Department of Biology, University of Nevada, Reno, 89557 Reno, USA; ^2^ Departamento de Ecología Evolutiva, Museo Nacional de Ciencias Naturales-CSIC, Madrid, 28006, Spain; ^3^ Department of Biology, University of Padova, 35131 Padova, Italy; ^4^ Institute of Avian Research, An Der Vogelwarte 21, 26386, Wilhelmshaven, Germany; ^5^ Department of Physiology, Veterinary School, Complutense University of Madrid, Avenida Puerta de Hierro s/n, 28040 Madrid, Spain; ^6^ Wageningen University and Research, 6708 Wageningen, The Netherlands; ^7^ Adam Mickiewicz University, PB61-712 Poznań, Poland; ^8^ National Biodiversity Future Center, 90133 Palermo, Italy

**Keywords:** parental care, sexual conflict, negotiation rules, repeatability, individual variation

## Abstract

Theoretical models indicate that the evolution of biparental care depends on how parents behaviourally negotiate their level of care in response to those of their partner and whether sexes and individuals consistently vary in their response (compensatory response). While the compensatory response has been widely investigated empirically, its repeatability has rarely been assessed. In this study, we used a reaction norm approach to investigate the repeatability of the compensatory offspring provisioning of a parent after temporary removal of its partner in the pied flycatcher (*Ficedula hypoleuca*) across different breeding seasons and partners. We found that only females partially compensated for the short-term removal of the partner and their response was significantly repeatable across years while breeding with different partners. This study highlights the importance of considering among individual differences in negotiation rules to better understand the role of negotiation mechanisms in the evolution of parental care strategies.

## 1. Introduction

Parental care strategies vary largely across animal species, ranging from biparental to no care [1]. Explanations for variation in patterns of care rely on the existence of a life-history trade-off between current and future reproduction [2,3] as well as on an evolutionary conflict of interests between the parents (sexual conflict) [4]. This evolutionary conflict is particularly evident in species with extended biparental care, and understanding how selection drives the evolution and persistence of biparental care systems has attracted the attention of evolutionary biologists.

Theoretical models indicate that biparental care is evolutionarily labile [5–7], influenced by several ecological and life-history factors [8–10], but also depends on how parents behaviourally interact with each other to continuously negotiate their level of care during a reproductive event [5]. In particular, a key prediction posits that biparental care can be evolutionarily stable if a decrease in parental investment by one parent is only partially compensated by the parental investment of its partner [11]. This prediction of ‘partial compensation’ has been tested empirically via experimental manipulation of partner's behaviour in a number of bird species [12]. These studies found substantial variation in the direction and magnitude of this compensatory response (for a summary on bird species see [13]), attributable to differences in the experimental set-ups [12,14], species [13], sexes [12,13] and parental tasks [15,16]. However, these studies have mostly explored variation in compensatory behaviour in terms of among-individuals (population mean) responses and have overlooked within-individual variation in responsiveness. Studies on within-individual variation are essential to understand how selection can act on parental phenotypes and on the evolution of parenting [17].

Investigating within-individual variation in parental behaviour has twofold applications. First, repeated measures of the same individuals enable us to quantify the plasticity of parental care in response to changes in socio-environmental conditions [17], and if it exhibits consistency [18]. Second, repeatable individual differences in parental care provide a first and simple estimation on variation and heritability of behaviours [19], which are required substrates for selection processes. Several studies have investigated repeatability of parental care behaviours such as provisioning rate [20–22], incubation [22,23] and predatory defence [24] across time and contexts, but to our knowledge repeatability of responses to changes in partner's contribution of parental provisioning has never been assessed before.

In this study, we used a reaction norm approach [18] to investigate across-year plasticity and repeatability of compensatory response, in terms of offspring provisioning, after temporary removal of the partner. Specifically, we aimed to investigate whether individual responses to partner removals were repeatable across different mates. The pied flycatcher, *Ficedula hypoleuca*, is an ideal system to investigate repeatability of parental behaviour: it is a palaearctic migratory species with high site fidelity for reproduction over multiple years [25]. Furthermore, both males and females usually breed with different mates across years [25]. This characteristic is relevant for our study, to distinguish the effect of the individuals from that of the pair, since pair bond duration and breeding experience with the same partner have been observed to alter parental behaviour [26,27].

## 2. Material and methods

### (a) Field methods

We used a breeding population of pied flycatcher situated near Valsaín, central Spain (40°53′74″N, 4°01′W, 1200 m.a.s.l.). This field site contains 300 nest-boxes and every year since 1991 an average of about 80 flycatcher pairs breed in this area. From the beginning of April, nest-boxes were routinely checked to monitor flycatcher reproduction and determine the onset of egg laying, incubation and hatching day (day 1).

In 2021, we experimentally tested 51 individuals (30 males, 21 females) from different nests. At day 10 of chick age, we recorded parental provisioning (no. of feeding trips) for 1 h (pre-removal section), starting between 07.30 and 08.00 h, using a video camera (Sony Handycam CX405) placed on the ground approximately 30 m from the nest. At the end of the pre-removal phase, we caught one parent with a trap (first individual that entered the nest) placed inside the nest-box. Capture order in this species is known to be age biased [28]. In this study, however, the focal and the caught parents did not statistically differ in age (paired *t*-test: *t*_50_ = −0.31, *p* = 0.758) and not in sex (exact binomial test: *p* = 0.262). We placed the caught individual inside a cotton bag until the end of the post-removal phase. One hour after the capture, we carried out another 1 h video recording session, in which we recorded the provisioning behaviour of the remaining parent (focal parent, post-removal phase). This allowed us to quantify the baseline provisioning rate of the two parents (feeding trips during the pre-removal phase) and the compensatory response of one parent after the reduction in provisioning rate by the other parent (feeding trips during the post-removal phase, hence compensatory response). At the end of the experimental protocol, the focal parents were captured and coloured-ringed to facilitate visual recognition in the following year. About 50% of the focal individuals tested in 2021 were found to breed in 2022 (16 males and nine females), and we repeated the same experimental procedure by removing their partner. In all cases, focal parents had a different partner from the previous season.

### (b) Statistical analyses

We first tested whether the total and individual feeding rate differed between sexes before and after partner removal. Linear mixed models (package ‘lme4’ [29]) were used, with treatment (pre versus post), sex and their interaction as fixed factors while controlling for brood size and hatching date. ‘Series ID’ (a series links the individual provisioning rates before and after removal in 1 year) nested in ‘Individual ID’ was included as a random structure. Repeatabilities of provisioning rates were estimated via two approaches. First, we used the R package ‘rptR’ [30] to calculate the repeatability of the focal parent's feeding rate before and after removal, separately for the two sexes. Second, we investigated whether parental responses to partner removal were repeatable by using a multi-level random regression reaction norm approach [31]. This method estimates the level of repeatability of the intercept and slope of parental provisioning rates in response to our experimental manipulation by quantifying variation in reaction norm intercepts and slopes within and among individuals [32]. Specifically, the intercept of an individual reaction norm corresponds to the provisioning rate before removal, whereas the slope represents the behavioural response following mate removal. Provisioning rates were modelled, separately for each sex, as a function of treatment (pre- versus post-removal). Random intercepts were included for individual and series; random slopes with respect to treatment were also included at two hierarchical levels [31]. We fitted the random regression models using a Bayesian framework implemented with the package ‘MCMCglmm’ [33]. Repeatabilities of slope and intercept were estimated as posterior means and 95% credible intervals (CIs). We deemed the slope and intercept of the parental response as repeatable if the lower CI was clearly away from zero [31,34]. Analyses were conducted in R v. 4.0 [35]. Provisioning rates were log_10_ transformed to improve normality of model residuals. For additional details about model implementations see the electronic supplementary material [36].

## 3. Results

**Figure 1.**
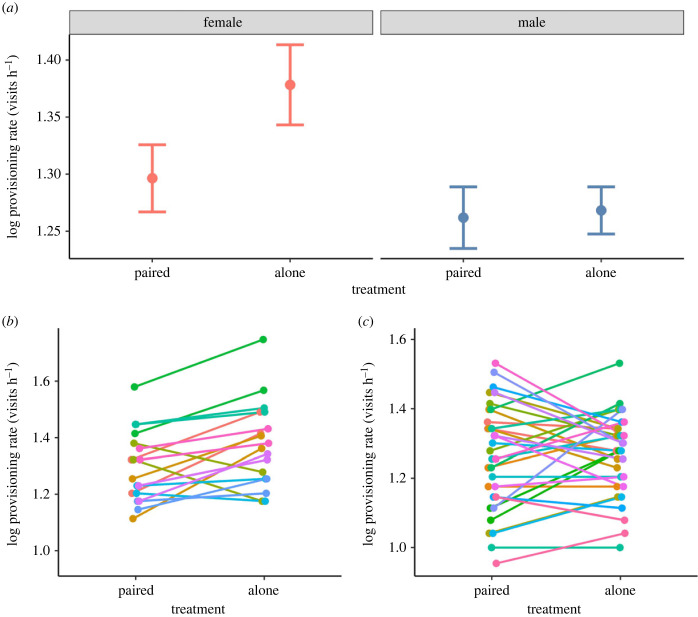
Provisioning rates of female and male parents before and after mate removal (*a*). Individual reaction norms for females (*b*) and males (*c*) in response to experimental treatment. Colours represent the single individuals. Mean ± s.e. is shown in (*a*).

The total provisioning rate at the nest significantly decreased between treatment phases (electronic supplementary material, table S1). Specifically, the provisioning rate of the focal parent differed between treatments in interaction with sex (*F*_1,48_ = 4.80, *p* = 0.033): only female parents but not males significantly increased their provisioning after mate removal (figure 1*a*, table 1). In addition, only females' but not males’ provisioning rates were repeatable across years when caring in pairs or alone (table 2). This result was also supported by the reaction norm analyses: repeatabilities of reaction norm intercepts and slopes were significantly different from zero only in females (intercept: *R* = 0.78 [0.38, 0.99]; slope: *R* = 0.90 [0.56, 1.00], figure 1*b*) but not in males (intercept: *R* = 0.19 [0.00, 0.55]; slope: *R* = 0.27 [0.00, 0.88], figure 1*c*).

**Table 1.** Model estimates and s.e. of the effect of mate removal on parental provisioning rate. Significant *p*-values are in italics.

variable	estimate	s.e.	*t*-value	*p*-value
LMM for provisioning rate				
intercept	0.42	0.24	1.70	0.095
brood size	0.08	0.02	4.60	*<0.001*
hatching date	0.01	0.00	2.25	*0.030*
treatment (alone)	0.08	0.03	2.97	*0.005*
sex (male)	−0.03	0.04	−0.75	0.459
treatment (alone) × sex (male)	−0.07	0.03	−2.19	*0.033*

**Table 2.** Adjusted repeatability (R) of provisioning rates for male and female parents across two consecutive breeding years. Estimates were calculated using intra-class correlation coefficients with parametric bootstrapping to obtain 95% confidence intervals (CI). Significant *p*-values are in italics.

variable	sex	R (CI)	*p*-value
provisioning rate in pair	male	0.05 (0,0.56)	0.457
	female	0.76 (0.25, 0.94)	*0.016*
provisioning rate after mate removal	male	0.34 (0,0.74)	0.124
	female	0.76 (0.21, 0.95)	*0.005*

## 4. Discussion

We first tested whether males and females responded differently to an experimentally reduced feeding contribution of their partners. Second, we tested whether pied flycatcher parents showed a repeatable feeding rate in response to experimental mate removal when mated with different partners in two successive breeding seasons. We found that only females increased their provisioning rates, partially compensating for the short-term removal of their partner. The reaction norm analysis indicates that only females' but not males’ responses to partner absence were significantly repeatable across breeding attempts with different partners.

Sex differences in compensatory response have been widely found across species [16,37–39]. A possible explanation is that sexes differ in the adopted negotiation rules [40,41] or in the information they gather on partner behaviour [42]. Specifically, females are known to be more responsive than males to changes in offspring demand occurring on a short timescale [41]. It remains to be tested whether a longer experimental removal of the female may elicit a compensatory response in males [32]. Another proposed explanation for the observed sex differences is that sexes might experience different costs or opportunities to re-nesting in the same season [43]. Under this scenario, the best strategy for females is to be responsive and keep investing in the current brood, whereas males would be better off adopting a stable investment strategy within the same breeding event [43]. However, this latter explanation is unlikely to apply in our system. The pied flycatcher is a single-brooded species (except in case of nest failure), and polygyny is rare in our population (3–4% of males, no experimental males were polygynous in this study).

Our across-year experimental set-up testing negotiated responses on the same individuals in a species with seasonal monogamy, allowed us to test the theoretical prediction that an individual parent's response to its partner reduction in provisioning rate was significantly repeatable across different partners [42,44]. In particular, our reaction norm approach enabled us to estimate the repeatability of the basal provisioning rate and the behavioural response to partner removal. Repeatability of baseline provisioning rates is well known both within-season and among-years [20–22,45] and sex differences in this repeatability is in line with another study on pied flycatchers [45]. No previous studies, however, have looked at repeatability of negotiation rules when the investment of the partner is experimentally reduced. In this regard, we found that only females' responses are repeatable over successive partners. Current negotiation models differ in the extent to which within- and among-individual variation in responsiveness is expected. In Lessells & McNamara's model [46] parents indirectly assess each other's investment by assessing the offspring state. This latter can vary across reproductive events and therefore an individual's negotiation response is expected to vary over time, i.e. compensation should not be repeatable [46]. By contrast, if the outcome of negotiation rules depends on individual differences in costs and benefits of provisioning [44], or in the accuracy to monitor partner's behaviour [44] and offspring condition [42], significant repeatability of negotiation responses is expected. More generally, cooperative models have emphasized the need for substantial among-individual differences in negotiation rules for stability of cooperation [47,48]. These conclusions seem particularly relevant considering that the pied flycatcher is socially monogamous, raises one brood per year but breeds with different partners over successive years. Parents are therefore under strong exploitation risk [11] and negotiated responses to partner's reduction in offspring provisioning are expected to be under strong selection [42]. Importantly, in this same sample, neither brood size nor hatching date were repeatable across years (see electronic supplementary material information), indicating that the repeatability of female provisioning rate and compensatory response was not the consequence of inter-individual variation in the reproductive investment/value. Our results therefore support theoretical models that predict that individual variation in negotiation rules facilitate evolutionary stable cooperation strategies in biparental species [49,50].

A limitation of the current study is that we simulated experimentally a single, short reduction in the provisioning rate of the partner. It may be possible that a response of the males to female removal may become evident after repeated or longer removal experiments. There are also other further experiments that may help elucidate negotiation dynamics. For example, one could look at the within-year (and within-pair) repeatability to see if parents are consistent or plastic in their response to multiple reductions of parental efforts over time. This can offer insights into how negotiation and investment rules could change over time [46], and how cooperation over parental care could be maintained [47,50] or break up [51] over repeated interactions. A second study could look at repeatability of negotiation rules in lifetime monogamous species. In this scenario, given that lifetime monogamy aligns the reproductive interests of the two parents [1], sexual conflict is reduced and therefore responsive rules between parents could change over time [44].

We encourage the concomitant use of experimental manipulation of parental effort and reaction norm approaches to estimate the within- and across-pair consistency or plasticity in responsiveness rules. We highlight the importance of considering among individual differences in negotiation rules to better understand the role of negotiation mechanisms in the evolution of parental care strategies.

## Data Availability

Data and R code are available from the Dryad Digital Repository: https://doi.org/10.5061/dryad.dfn2z3576 [36]. The data are also provided in the electronic supplementary material [52].
